# MiR‐375/SLC7A11 axis regulates oral squamous cell carcinoma proliferation and invasion

**DOI:** 10.1002/cam4.1110

**Published:** 2017-06-19

**Authors:** Yadong Wu, Xiangjie Sun, Bin Song, Xiaoling Qiu, Jianjiang Zhao

**Affiliations:** ^1^ Department of Oral and Maxillofacial Surgery Stomatological Hospital of Southern Medical University & Guangdong Provincial Stomatological Hospital Southern Medical University Guangzhou 510260 Guangdong China; ^2^ Department of Stomatology Guizhou Provincial People's Hospital Guiyang 550002 Guizhou China; ^3^ Department of Pathology Fudan University Shanghai Cancer Center Shanghai 200032 China; ^4^ Department of Oncology Shanghai Medical College Fudan University Shanghai 200032 China

**Keywords:** miR‐375, OSCC, SLC7A11

## Abstract

We aimed to detect the functions of miR‐375/SLC7A11 axis on oral squamous cell carcinoma (OSCC) cell proliferation and invasion. Expression levels of miR‐375 and SLC7A11 in OSCC tissues and cells were measured with RT‐qPCR and western blot. Targeting site was predicted by TargetScan and confirmed by dual luciferase reporting assay. By way of manipulating the expression level of miR‐375 and SLC7A11 in CAL‐27 and Tca8113 cell lines, the cell biological abilities were evaluated. MTT, colony formation, Transwell, wound healing assays and flow cytometry were used to detect OSCC cell viability, proliferation, invasion, migration and apoptosis, respectively. MiR‐375 was significantly downregulated in OSCC tissues and cells compared to adjacent tissue and normal oral cell line respectively while SLC7A11 was upregulated. Targeting relationship was verified by luciferase reporting assay, and miR‐375 could effectively suppress SLC7A11 level in OSCC cells. Replenishing of miR‐375 significantly repressed OSCC cell viability, proliferation, invasion and migration and induced cell apoptosis and G1/G0 arrest. Overexpression of SLC7A11 recovered those biological abilities in miR‐375 upregulated cells. Collective data suggested that miR‐375 served as a tumor suppressor via regulating SLC7A11. Replenishing of miR‐375 or knockout of SLC7A11 could be therapeutically exploited.

## Introduction

Oral squamous cell carcinoma (OSCC) is perhaps the most prevalent type of head and neck cancer and it accounts for nearly 90% malignant oral tumor cases 1, 2, 3. The 5‐year survival rate of OSCC patients is approximately 60–80% which is reduced to 50% if OSCC is not diagnosed timely 3, 4, 5. Meanwhile, the potential molecular mechanism of OSCC is not integrity and, more resources should be allocated to this area to discover the corresponding targeted therapy.

MicroRNAs (miRNAs) are a large set of small noncoding RNAs which are involved in the gene expression regulation 6. By base pairing to the 3'‐UTR (Untranslated regions) of mRNA, miRNAs can downregulate their target mRNAs at the posttranscriptional level 7, 8, 9. As suggested by several studies, miRNAs can function as biomarkers for the purpose of cancer screening, diagnosis and prognosis. For instance, Shin et al. reported that miR‐181a was downregulated in OSCC, indicating that miR‐181a may function as an OSCC suppressor 10. Sun et al. found that both miR‐328 and miR‐378 may trigger the brain metastasis of non‐small‐cell lung cancer; therefore they may function as predictive biomarkers for diagnosing cancer progression 11. Xiao et al. indicated that miR‐144 could suppress the proliferation and migration of colorectal cancer cells 11. The complex role of miRNA in tumors has been quite controversial in the current literature since some miRNAs were classified as tumor suppressors whereas others were classified as oncogenic miRNAs 12, 13. For instance, high miR‐21 expression was confirmed to be associated with the proliferation and invasion of breast cancer 14. On the contrary, low expressions of miR‐1 were observed in patients with bladder cancer, implying that restoring the expression of miR‐1 is a potential targeted therapy for bladder cancer patients 15. Therefore, clarifying various roles of miRNAs in different types of cancers may improve the corresponding results of cancer screening, diagnosis and prognosis.

As a member of miRNAs, miR‐375 has been reported to be associated with many cancers, including hereditary medullary thyroid cancer 16, hormonal breast cancer 16 and pancreatic cancer 17. Moreover, some exploratory researches with respect to the potential role of miR‐375 in OSCC have been carried out and these studies revealed a preliminary conclusion that miR‐375 may function as a tumor suppressor 1, 18. However, researches should be progressed further in order to justify these preliminary conclusions.

On the other hand, SLC7A11 (Solute carrier family seven number 11), also known as xCT, is the main component of system, x_c_^‐^ which plays a key role in the extracellular cysteine transportation 19, 20. Cysteine is the raw material of the intercellular synthesis of GSH (glutathione) and a reduced state of GSH can mediate cellular detoxification 19, 21. Growing evidence revealed that SLC7A11 was associated with preneoplastic lesions and cancer 1, 22, 23, 24. The expression of SLC7A11 has been detected in multiple malignant tumors and can be linked with poor prognosis and medication resistance 25, 26, 27. Apart from the potential relationship between SLC7A11 and ovarian cancer, another study discovered that the expression of SLC7A11 is able to regulate the resistance of cisplatin in patients with tongue squamous cell carcinoma which is another type of oral tumor 19, 28.

Since SLC7A11 is one target gene of miR‐375, we suspected that miR‐375 is likely to modulate the expression of SLC7A11. This study was carried out in order to link both miR‐375 and SLC7A11 with OSCC. For this purpose, multiple experimental protocols were designed and conducted: RT‐qPCR, Western Blot and Luciferase experiment. The above experiments confirmed our hypothesis that miR‐375 can target SLC7A11 and miR‐375 can function as a tumor suppressor for OSCC. More importantly, miR‐375 inhibited the proliferation and invasion of OSCC through suppressing the expression of SLC7A11.

## Materials and methods

### Clinical specimens

Oral squamous cell carcinoma sample tissues and matched adjacent tissues (at least 2 cm distal to the tumor margin) were obtained from 40 patients who were admitted to Stomatological Hospital of Southern Medical University and Guangzhou Provincial People's Hospital during 4/2015 to 7/2016. None of the OSCC patients had received adjuvant radiotherapy or chemotherapy before surgery. All samples were verified by experienced pathologists and then frozen at once in liquid nitrogen at −80°C. The study gained approval from the Institutional Review Board of Stomatological Hospital of Southern Medical University and Guangzhou Provincial People's Hospital, while each patient provided written informed consents.

### Cell lines and cell culture

Normal human oral cell line Hs 680.Tg and four human OSCC cell lines, including Fadu, SCC‐25, CAL‐27 and Tca8113, were cultured in DMEM containing 10% FBS. All cell lines were purchased from the American Type Culture Collection (ATCC, Manassas, VA) and maintained in the incubator with 5% CO_2_ at 37°C.

### RNA isolation and RT‐qPCR analysis

The total RNA was extracted using QIAzol reagent (Qiagen, Duesseldorf, Germany) and cDNA was synthesized, using a cDNA reverse transcription kit (Fermentas, Waltham, MA). RT‐qPCR was performed using SYBR‐Green PCR kit (Invitrogen, Carlsbad, CA) following the manufacturer's procedure. U6 snRNA was used to normalize miR‐375 while GAPDH was used to normalize SLC7A11 mRNA. The relative expression levels of miR‐375 and SLC7A11 were calculated using $2^{-\Delta \Delta c_{t}}$ method. The designed RT‐qPCR primers were shown in Table 1.

**Table 1:** Primers designed for RT‐qPCR

	Forward primer sequences	Reverse primer sequences
miR‐375	5'‐GTGCAGGGTCCGAGGT‐3'	5'‐AGCCGTTTGTTCGTTCGGCT‐3'
SLC7A11	5'‐GCTGTGATATCCCTGGCATT‐3'	5'‐GGCGTCTTTAAAGTTCTGCG‐3'
U6	5'‐CTCGCTTCGGCAGCACA‐3'	5'‐AACGCTTCACGAATTTGCGT‐3'
GAPDH	5'‐ACAACTTTGGTATCGTGGAAGG‐3'	5'‐GCCATCACGCCACAGTTTC‐3'

### Western blot analysis

Cells were lysed with CelLytic M Cell Lysis Reagent (Sigma‐Aldrich, St. Louis, MO) for total protein extraction. Protein concentrations were quantified by Bradford method. The proteins were separated by a 10% SDS‐polyacrylamide gel and eleco‐transferred onto PVDF membranes (GE Healthcare, Little Chalfont, Buckinghamshire, UK), which were incubated in 5% skim milk for 1.5 h at room temperature. Primary antibodies against SLC7A11 (Sigma‐Aldrich, catalog number: SAB2900091) and GAPDH (Sigma‐Aldrich, catalog number: SAB2701826) were diluted at 1:1000 and then incubated with the membranes overnight at 4°C. The next day HPR‐conjugated secondary antibodies were added to the membranes and incubated for 1 h. The integral optical density value of the protein bands was determined and quantified, using Fusion SL imaging system (Viber Lourmat, Marne‐ La‐Valee, France). GAPDH was used as an internal control and the experiments were repeated three times.

### Cell transfection

Cells in logarithmic phase were collected and seeded into 6‐well plates (1 × 10^6^/well). When reaching 80% confluence, cells were transfected with 100 nmol/L miR‐375 mimics, 100 nmol/L miR‐375 mimics control, 100 nmol/L SLC7A11 siRNA, 1.0 μg pCDNA3 or 1.0 μg pCDNA3.1‐SLC7A11 using Lipofectamine^TM^ 2000 (Invitrogen) following the manufacturer's procedure. The cells were harvested 48 h after transfection. The miR‐375 mimics, miR‐375 NC, SLC7A11 siRNA and SLC7A11 cDNA were synthesized by Shanghai Gene Pharma co., Ltd (Shanghai, China).

### Dual luciferase reporter assay

PmiRGLO Dual Luciferase miRNA Target Expression Vector (E1330; Promega, Madison, WI) was used to construct the wild type and mutated type of SLC7A11 3'UTR. While the wild‐type 3'UTR of SLC7A11 is complementary to the seed region of miR‐375 (GAACAAA; nt 482–488), the mutated type 3'UTR is badly complementary to the seed region of miR‐375. MiR‐375 mimics or miR‐375 NC (50 nmol/L) and wild type or mutated type of SLC7A11 3'UTR vectors (500 ng) were co‐transfected into HEK293T cells (ATCC, Manassas, VA) using Lipotectamine^TM^ 2000 (Invitrogen) following the manufacturer's instructions. The luciferase activities were quantified by Dual‐Luciferase Reporter Assay System (Promega). Relative luciferase activities were calculated by firefly luciferase activities/Renilla luciferase activities.

### MTT assay

Cells collected in the logarithmic phase were plated into 96‐well plates (2 × 10^3^ cells/well). 20 μL of 5 g/L MTT solutions (Sigma, Saint Louis, MO) were added into each well daily over a 3‐d time course and incubated for 4 h every day, followed by the addition of 150 μL DMSO and incubated for another 15 min. The optical absorbance was measured at the wavelength of 570 nm.

### Colony formation assay

After 24 h of transfection, cells were seeded in 96‐well plates (2 × 10^3^ cells/well) until colonies were visible (nearly 12 days). The colonies were then fixed with methanol and stained with 0.25% crystal violet for 30 min. The number of the colonies was then counted with naked eyes.

### Transwell assay

Cells were digested into single cell suspension (5 × 10^4^ cells/mL) after 48 h of incubation. The upper Transwell chambers (8.0 μm pore size; Merck Millipore, Billerica, MA) were coated with Matrigel (5× dilution; 20 μL/well; BD Biosciences, San Jose, CA) while the lower ones were added with 1 mL cell culture with DMEM containing 10% FBS. Cells on the upper layer of the membrane were removed and those migrated through the membrane were fixed and stained 24 h after the incubation. At least four fields of each membrane were observed and photographed under a microscope. The number of invading cells was presented as the average number of cells in every microscopic fields of every well.

### Wound healing assay

Straight lines were drawn on the back of 6‐well plates using a marker pen. Cells were seeded into six‐well plates (5 × 10^5^ cells/well). Wounds were then created, using a 200 mL pipette tip. The scratched cells were removed by the PBS for three times. After 24 h of incubation, the wound healing condition was photographed under an optical microscope and the wound closure was analyzed.

### Cell cycle analysis

After 48 h of incubation, transfected cells were digested with trypsin and centrifuged at 1410 g. The deposition was re‐suspended with PBS, thereafter the cell suspension was fixed by 75% ethanol on ice for 12 h. Cells were incubated in a dark room for 30 min after the addition of 100 μL PI and 100 μL RNA enzyme solutions. Cell cycle progression was analyzed based on FACS method using a Flow Cytometer (BD Biosciences).

### Apoptosis analysis

After 48 h of incubation, transfected cells were digested by trypsin and centrifuged at 1000 rpm. The sedimentary cells were resuspended and adjusted at the density of 1 × 10^6^ cells/mL. Cell suspension was incubated in a dark room for 15 min after the addition of 5 μL Annexin‐V‐FITC and 5 μL PI solutions. Then flow Cytometer was used to analyze the cell apoptosis.

### Statistical analysis

All statistical data were analyzed, using SPSS (Version 21.0; IBM, Armonk, NY) and figures were constructed using GraphPad Prism 6.0. Measurement data were presented as mean ± standard deviation (SD) and analyzed, using Student's *t*‐test (only two groups) or one‐way ANOVA (more than two groups) if they complied with normal distribution. Otherwise, Mann–Whiney was used. *P *< 0.05 was considered as statistically significant.

## Results

### MiR‐375 was downregulated in human OSCCs tissues while SLC7A11was upregulated

**Figure 1:**
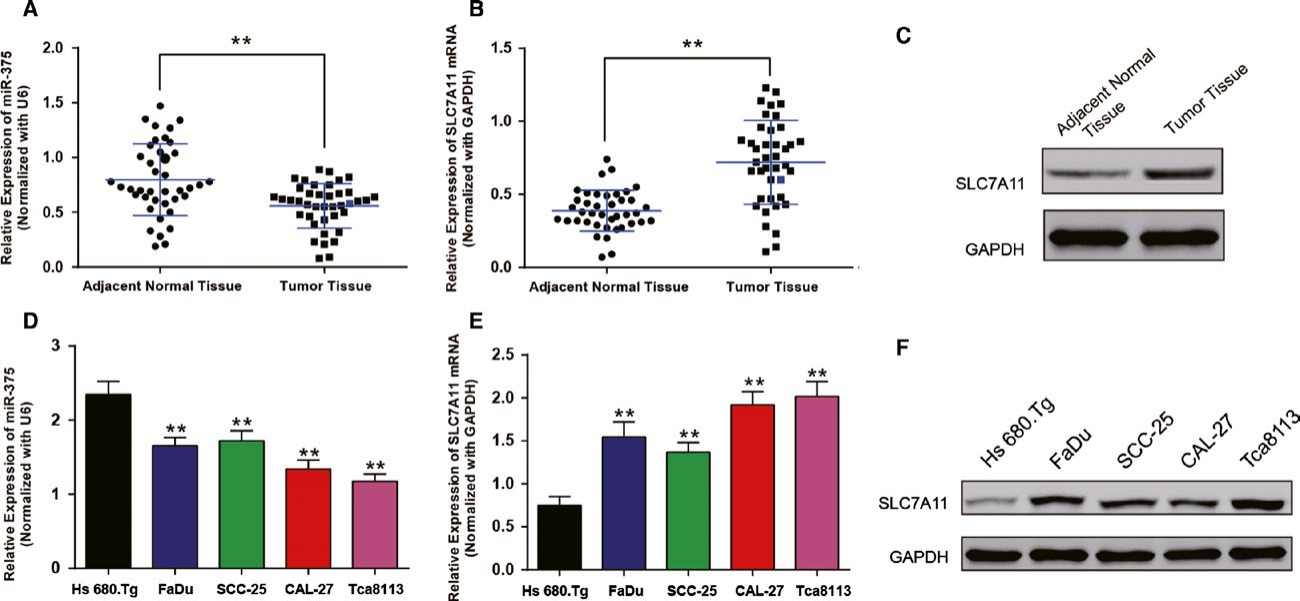
MiR‐375 was downregulated in human oral squamous cell carcinoma (OSCC) tissues and cells while SLC7A11 was upregulated. (A) The relative expression level of miR‐375 in 40 paired OSCC tissues and adjacent normal tissues, *^*^ *P *<* *0.05 versus adjacent normal tissues. (B) The relative expression of SLC7A11 mRNA in 40 paired OSCC tissues and adjacent normal tissues, *^*^ *P *<* *0.05 versus adjacent normal tissues. (C) The expression of SLC7A11 protein in OSCC tissues and adjacent normal tissues. (D) The relative expression level of miR‐375 in human OSCC cell lines and normal oral cell line (Hs 680.Tg), *^*^ *P *<* *0.05 versus Hs 680.Tg. (D) The relative expression of SLC7A11 mRNA in normal oral cell line and human OSCC cell lines, *^*^ *P *<* *0.05 versus Hs 680.Tg. (E) The expression of SLC7A11 protein in human OSCC cell lines and normal oral cell line, GAPHD acted as an internal control.

Inspired by previous studies and GCTA database, we sought to identify the expression level of miR‐375 and SLC7A11 in OSCC tissues and cells with RT‐qPCR and western blot assay. In 40 paired OSCC tissues and adjacent normal tissues, MiR‐375 expression frequently decreased in the OSCC tissues compared with adjacent tissues while SLC7A11 expression was upregulated at both mRNA and protein levels (*P *<* *0.05; Fig. 1A–C).

RT‐qPCR and western blot were also performed in normal human oral cell line Hs 680.Tg cell line and four different human OSCC cell lines including FaDu, SSC‐25, CAL‐27 and Tca8113. As shown in Figure 1D, miR‐375 expression dramatically decreased in the four cancerous cell lines compared with Hs 680.Tg cell line (*P *<* *0.05). And in Figure 1E–F, the mRNA expression and protein level of SLC7A11 increased in the four cancerous cell lines compared with Hs 680.Tg cell line (*P < *0.05). CAL‐27 and Tca8113 cells were then selected for subsequent experiments.

### MiR‐375 directly targeted SLC7A11

**Figure 2:**
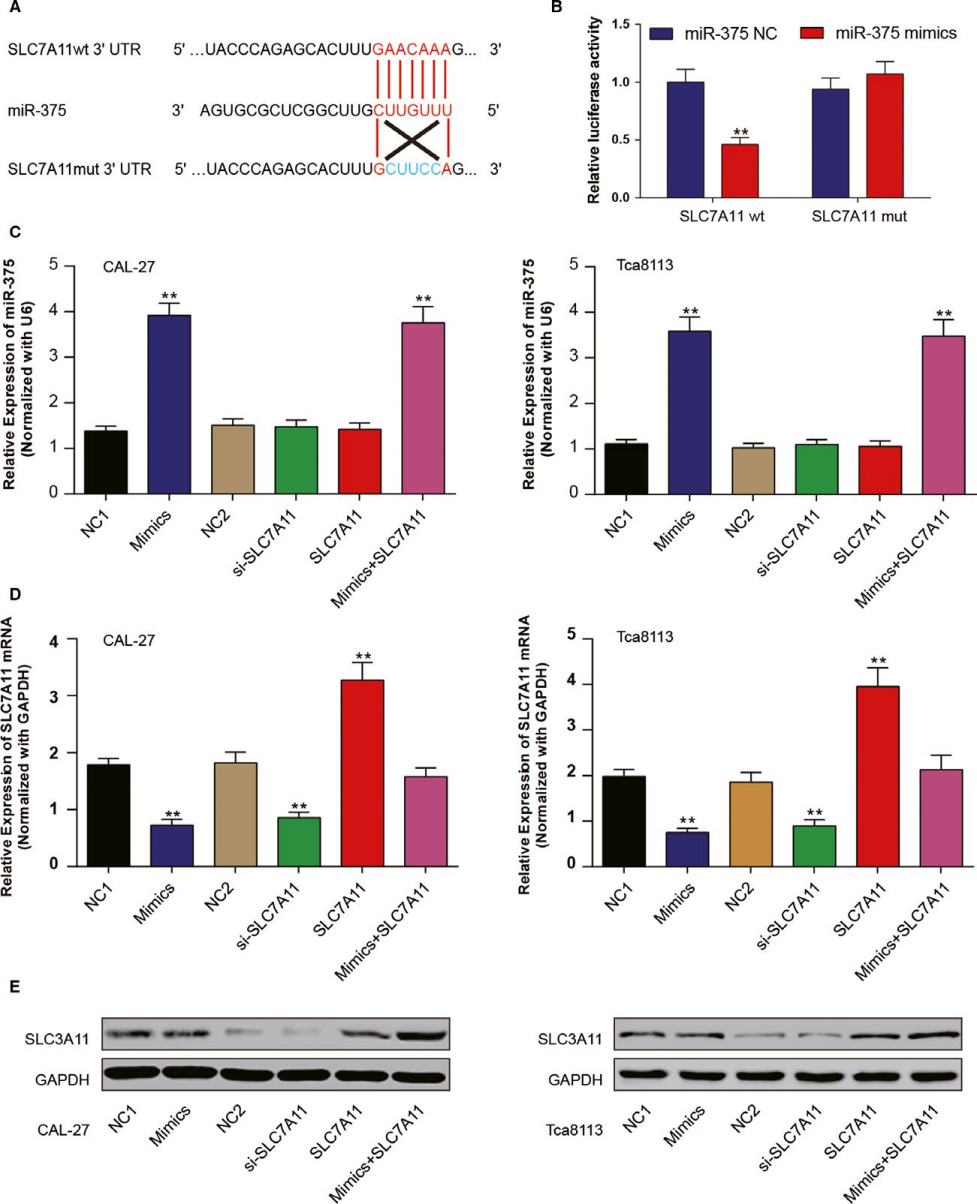
MiR‐375 directly targeted SLC7A11. (A) 3'UTR region of SLC7A11 mRNA is partially complementary to miR‐375. (B) The luciferase activities of HEK293T cells in different groups. (C) The expression of miR‐375 in transfected cells was measured by RT‐qPCR. (D–E) The mRNA expression and protein level of SLC7A11 was evaluated by RT‐qPCR and western blot respectively. *^*^ *P *<* *0.05. All data was presented as Mean ± SD from three independent experiments.

According to the prediction by TargetScan database, SLC7A11 3'UTR contains a putative miR‐375 target site. The Figure 2A illustrated the corresponding sequences of miR‐375, wild type SLC7A11 3'UTR and mutation of SLC7A11 3'UTR. Ectopic expression of miR‐375 suppressed the luciferase activities of plasmids containing wild type SLC7A11 3'UTR but had no effect on the PmiRGLO‐SLCA11 mutant 3'UTR (*P < *0.05, Fig. 2B).

Further transfection was carried out in CAL‐27 and Tca8113 cells. Cells were divided as six groups: NC1 group transfected with 100 nmol/L miR‐375 mimics control, Mimics group transfected with 100 nmol/L miR‐375 mimics, NC2 group transfected with 1.0 μg pCDNA3.1 vector, si‐SLC7A11 group transfected with 100 nmol/L SLC7A11 siRNA, SLC7A11 group transfected with 1.0 μg pCDNA3.1‐SLC7A11, mix group transfected with 100 nmol/L mir‐375 and 1.0 μg pCDNA3.1‐SLC7A11. At 48 h after transfection, cells were collected and following assays were performed. RT‐qPCR and western blot results confirmed the efficiency of transfection in CAL‐27 and Tca8113 cells (Fig. 2C–E). MiR‐375 increased in mimics group and mix group compared with negative control group while the expression of SLC7A11 increased in SLC7A11 group and decreased in mimics group and si‐SLC7A11 group. Moreover, in mix group, replenishing of pCDNA3.1‐SLC7A11 recovered the expression of SLC7A11 which was originally repressed by miR‐375. In total, miR‐375 was verified to target SLC7A11 3' UTR and suppressed its expression.

### MiR‐375/SLC7A11 axis functions on OSCC cell viability and proliferation

To investigate the effects of miR‐375/SLC7A11 on cell proliferation, CAL‐27 and Tca8113 cells were first transiently transfected with miR‐375 mimics, SL7A11 siRNA or pCDNA3.1‐SLC7A11, respectively at different concentration. Cell viabilities were measured by MTT assay and assessed as a ratio of OD value compared with cells treated with same amount of Lipofectamine 2000 reagents at 72 h after transfection. As shown in Figure S1, once the concentration of miR‐375 reached 50 nmol/L or the concentration of SLC7A11 siRNA reached 75 nmol/L, cell viabilities were significantly inhibited (*P *<* *0.05, Fig. S1A–B). Meanwhile, pCDNA3.1‐SLC7A11 remarkably promoted cell growth at a concentration of more than 0.5 μg (*P *<* *0.05, Fig. S1C). To increase the difference, the final transfection concentration of miR‐375 mimics control, miR‐375 mimics, SLC7A11 siRNA was 100 nmol/L, while the pCDNA3.1 vector and pCDNA3.1‐SLC7A11 were utilized at 1.0 μg. In mimics group, cells were transfected with 100 nmol/L miR‐375 and 1.0 μg pCDNA3.1‐SLC7A11.

**Figure 3:**
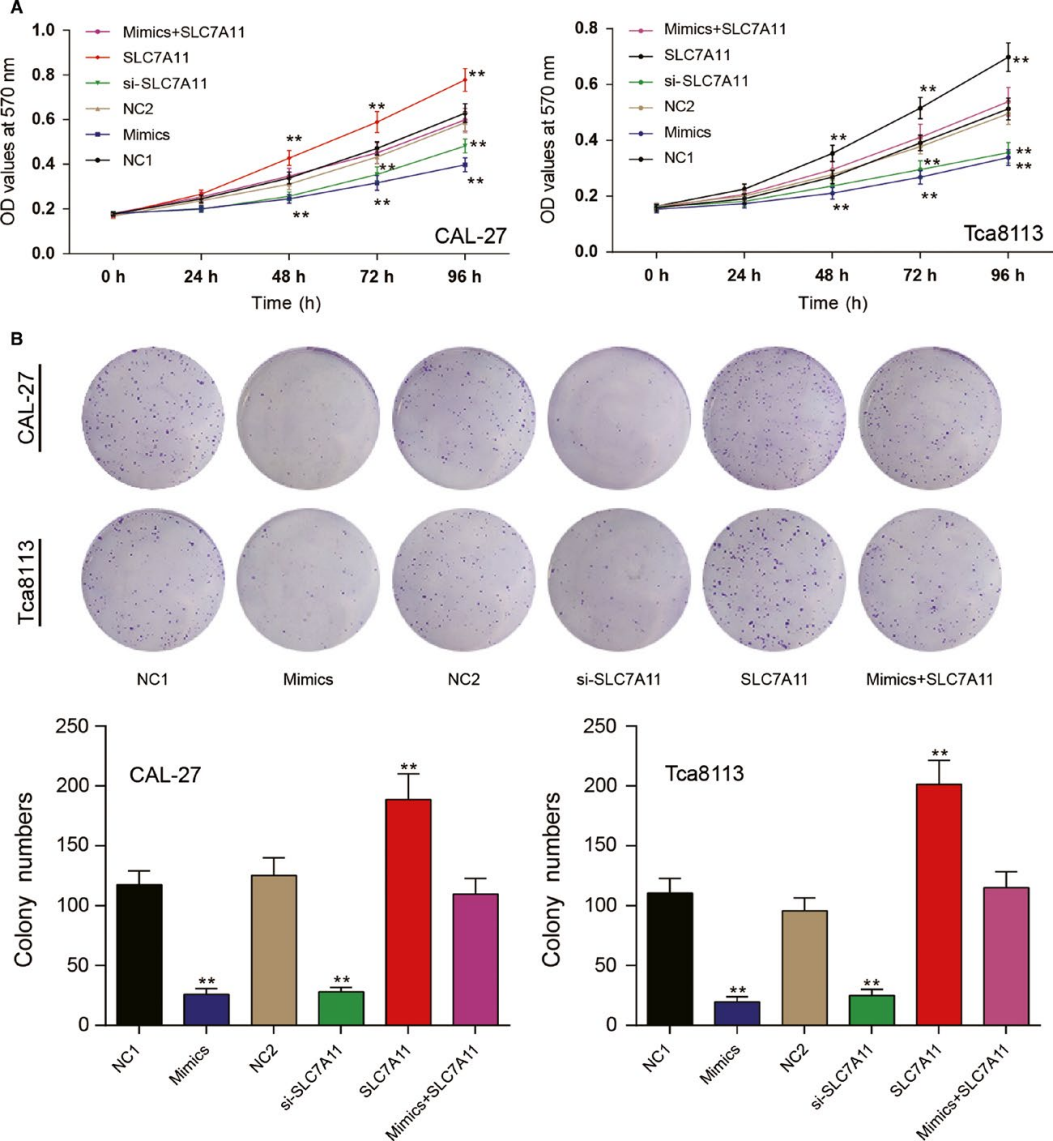
The effects of miR‐375/SLC7A11 axis on oral squamous cell carcinoma (OSCC) cell viability and proliferation. (A) MTT assay was performed to assess cell viability. MiR‐375 and SLC7A11 siRNA significantly lightened OD value of CAL‐27 and Tca8113 cells while pCDNA3.1 accelerated. (B) The effects of miR‐375 and SLC7A11on OSCC cell growth were further confirmed by colony formation. *^*^ *P *<* *0.05 versus NC1 group. All data was presented as Mean ± SD from three independent experiments.

The growth curve of transfected CAL‐27 and Tca8113 cells were shown in Figure 3A, no difference was seen among the NC1 group and the NC2 group. MiR‐375 dramatically suppressed OSCC cell viability. Compared with negative control groups, the absorbance at 570 nm significantly decreased in cells in miR‐375 mimics or siRNA‐SLC7A11 group after 72 h (*P < *0.05), whereas cells transfected with pCDNA3.1‐SLC7A11 exhibited showed increased OD values. In mix group, replenishing of pCDNA3.1‐SLC7A11 recovered the cell growth and no significant difference was analyzed compared with NC groups.

Further, the effects of miR‐375/SLC7A11 on OSCC cell growth was confirmed by colony formation assay (Fig. 3B). Compared with negative control groups, the number of colonies in mimics and si‐SLC7A11 groups were much less. However, upregulation of SLC7A11 facilitated colony formation and could reverse the inhibition of miR‐375 mimics in mix group. Collective data demonstrated that miR‐375 suppressed OSCC cell proliferation and viability via downregulating SLC7A11 expression.

### MiR‐375/SLC7A11 axis functions on OSCC cell invasion and migration

**Figure 4:**
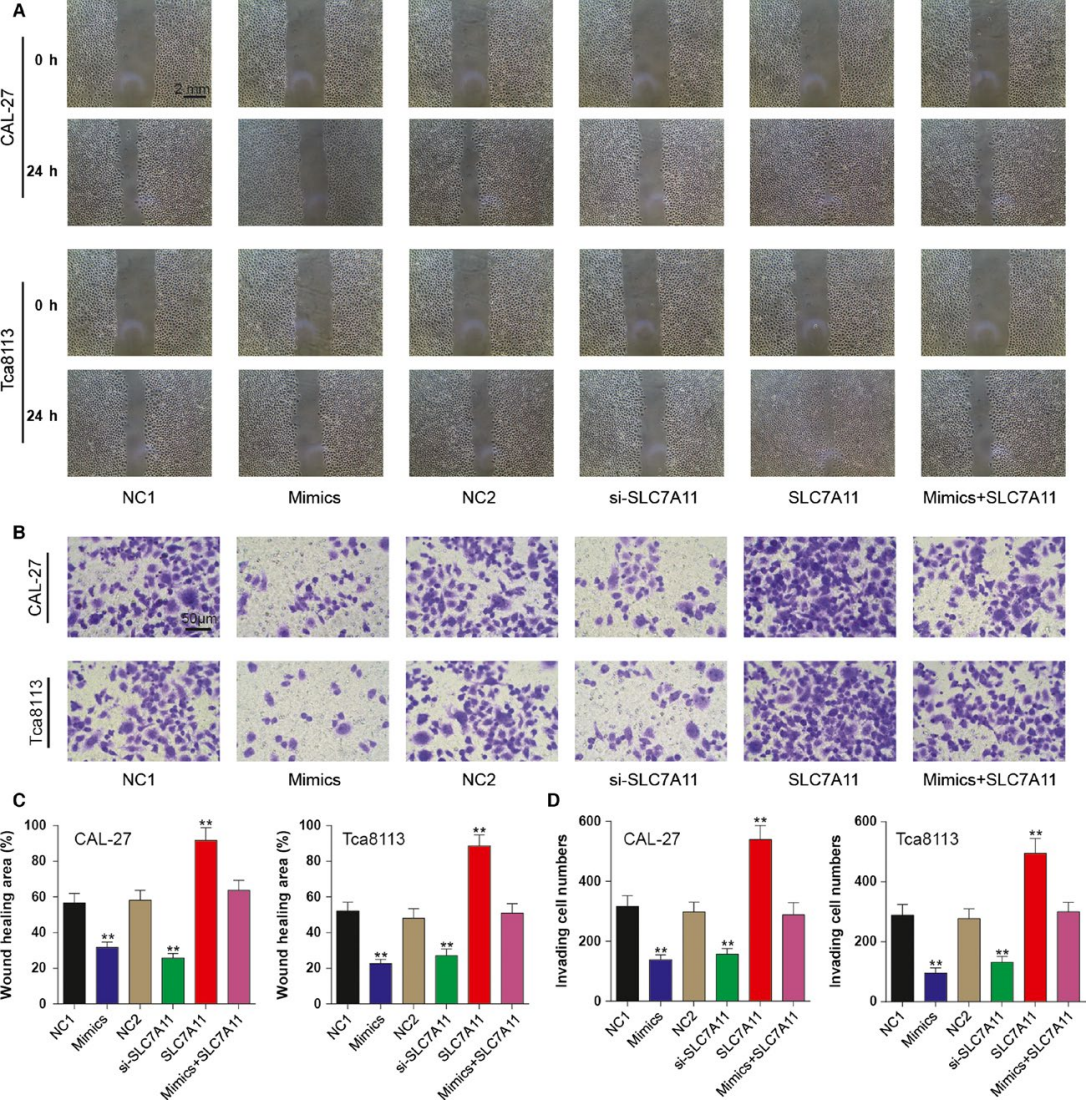
The effects of miR‐375/SLC7A11 axis on oral squamous cell carcinoma cell invasion and migration. (A) At 48 h after transfection, wound healing assay was performed to assess the migration ability of CAL‐27 and Tca8113 cells. (B) At 48 h after transfection, Transwell assay was utilized and the influence of miR‐375/SLC7A11 axis on cell invasive abilities was investigated. *^*^ *P *<* *0.05 versus NC1 group. All data was presented as Mean ± SD from three independent experiments. (C) Wound healing area of all groups was calculated with the help of Adobe Photoshop CS6. (D) After staining the invading cells, cell number of five random fields was calculated and compared. ** *P* < 0.05 vs. NC1 group. All data was presented as Mean ± SD from three independent experiments.

Transwell assay demonstrated that the number of cells that invaded through matrigel matrix frequently decreased in cells (CAL‐27 and Tca8113 cells) transfected with miR‐375 mimics or SLC7A11 siRNA but increased in cells transfected with pcDNA3.1‐SLC7A11 compared with the corresponding NC groups (*P < *0.05; Fig. 4A). And a similar phenomenon was observed by wound healing assay (Fig. 4B). Cells in the mimics or si‐SLC7A11 group presented a smaller closure rate than those in NC1 and NC2 groups, whereas those in SLC7A11 group manifested bigger closure rate. Cells in mix group acted a medium migration and invasion ability which was close to negative groups. All evidences indicated that the regulation of miR‐375 on cell invasion and migration was via inhibiting SLC7A11.

### MiR‐375/SLC7A11 axis functions on OSCC cell mitosis and apoptosis

**Figure 5:**
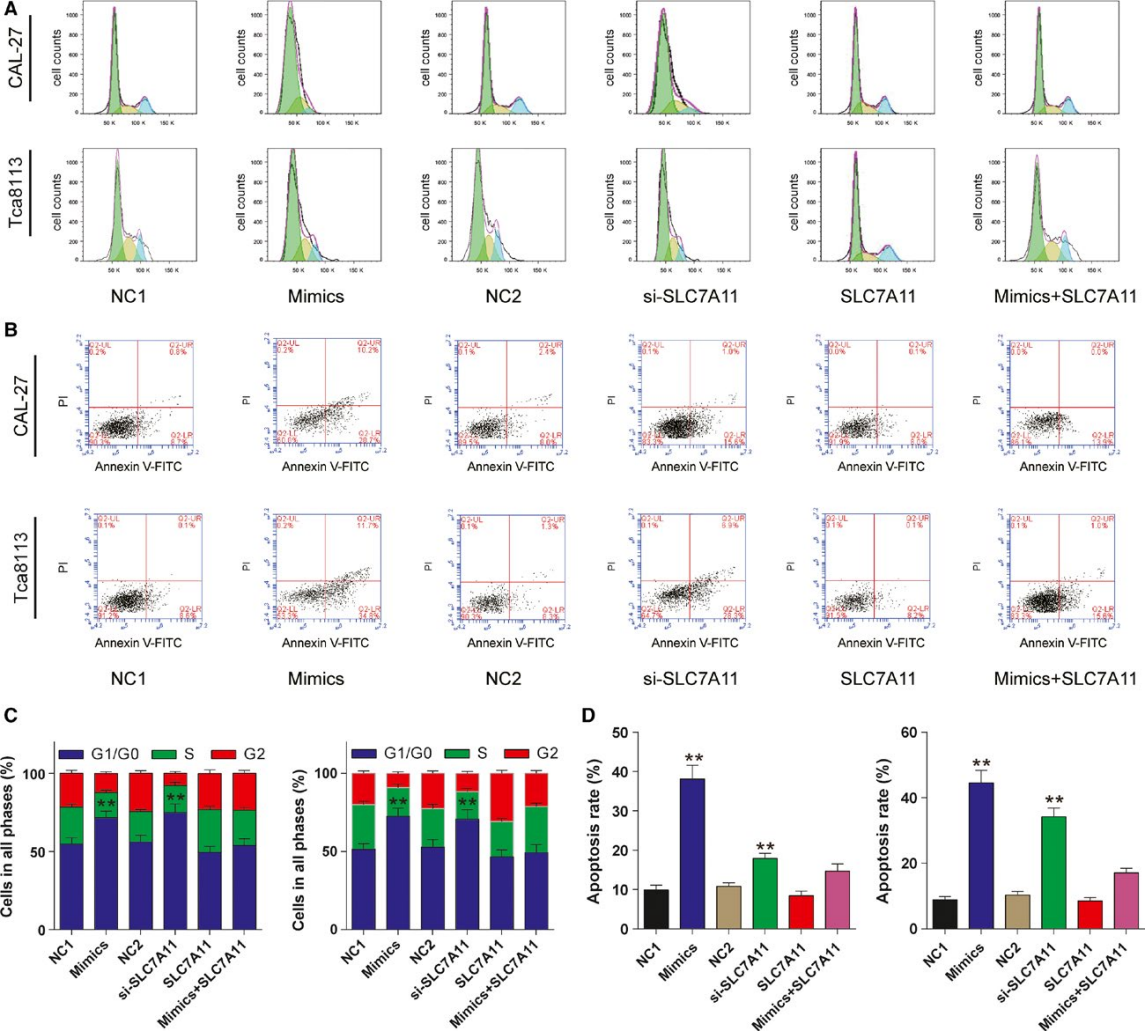
The effects of miR‐375/SLC7A11 axis on oral squamous cell carcinoma cell mitosis and apoptosis. (A) At 48 h after transfection, PI staining was performed and flow cytometry was utilized to count cells in each phase. G1/G0 arrest was confirmed in cells transfected with miR‐375 mimics and SLC7A11 siRNA. (B) At 48 h after transfection, cells were stained with PI and Annexin V‐FITC and separated by flow cytometry. Cell apoptosis was induced in mimics group and si‐SLC7A11 group. *^*^ *P *<* *0.05 versus NC1 group. All data was presented as Mean ± SD from three independent experiments. (C‐D) Cell cycle and apoptosis was analyzed with FlowJo_V10 and collective data was compared. **P < 0.05 vs. NC1 group. All data was presented as Mean ± SD from three independent experiments.

To further investigate the role of miR‐375/SLC7A11 on OSCC cell mitosis and apoptosis, flow cytometry was used with PI and Annexin V‐FITC staining. As shown in Figure 5, the addition of miR‐375 mimics or SLC7A11 siRNA contributed to the cell cycle arrest in G0/G1 phase and induced cell apoptosis compared to negative control groups. Though overexpression of SLC7A11 has little impact on cell cycle and apoptosis compared with negative control groups, it could lighten the G0/G1 arrest and decrease apoptosis rate in mix group. Taken together, we demonstrated that miR‐375 induced G1/G0 arrest and accelerated cell apoptosis via downregulating SLC7A11.

## Discussion

Oral squamous cell carcinoma is a high‐risk cancer that afflicts a large number of populations and it threats the life of patients by local recurrence and metastatic neck lymph nodes 1, 2, 3, 29, 30. The complex mechanisms of OSCC were widely studied and we found that SLC7A11 was upregulated in OSCC cells while the expression of miR‐375 exhibited the opposite trend. When the expression of miR‐375 was intentionally elevated artificially, the corresponding activity of OSCC was suppressed. Moreover, miR‐375 can negatively regulate SLC7A11, suppressing the invasion and proliferation of OSCC cells.

A large number of reports have suggested the potential role of miR‐375 in colorectal carcinoma, head and neck squamous cell cancer and prostate cancer 31, 32, 33. As suggested by the majority of these studies mentioned above, miR‐375 was classified as a significant tumor suppressor and increasing evidence supported the role of miR‐375 in inflammation and cancers 1, 34, 35. For instance, Kozaki et al. discovered that 36.5% miRNAs were downregulated in OSCC cell lines 12. Though there is a few reports focusing on the relationship between miR‐375 and OSCC, indicating that miR‐375 may react as a suppressor, it is still essential to obtain further evidence 1, 36, 37, 38. In our study, the expression of miR‐375 was downregulated in OSCC cell lines and the proliferation and invasion of OSCC cells were inhibited when we elevated the expression of miR‐375.

MiRNAs are known to act their functions by repressing their target genes, binding to the 3'UTR (untranslated region) of their target genes mRNA 39. Shi et al. reported that SLC7A11 may be one of the most important target genes of miR‐375 1. Predicted by TargetScan and verified by dual luciferase reporting assay, SLC7A11 was confirmed to be a direct target of miR‐375. Moreover, replenishing of miR‐375 decreased both mRNA expression and protein level of SLC7A11 in OSCC cells. SLC7A11 is one of the amino‐acid transporters, which plays an important role in cell metabolism 19, 20. Previous studies indicated that SLC7A11 plays a key role in chemotherapy resistance 28, 40. A study conducted by Zhang et al. reported that SLC7A11 may modulate cisplatin (CDDP) resistance and they also concluded that CDDP can elevate the expression of SLC7A11 significantly in TSCC cells (tongue carcinoma cells) which is another type of head and neck tumor 28. However, Zhang et al. did not study the corresponding mechanism of SLC7A11 at miRNA levels which may impact on the proliferation, invasion and metastasis of OSCC. Results of Western blot indicated that SLC7A11 was upregulated in OSCC cells and silencing of SLC7A11 resulted in an inhibition in the proliferation and invasion of OSCC cells as well as miR‐375 mimics. Therefore, we assumed that miR‐375 functions as a tumor‐suppressing role in OSCC via downregulating SLC7A11 expression.

Recovery experiment was performed by overexpressing miR‐375 and SLC7A11 in CAL‐27 and Tca8113 cells simultaneously. The evaluating level of SLC7A11 reversed the tumor‐suppressing effect of miR‐375 on cell proliferation, migration, invasion, mitosis and apoptosis, which further confirmed the important role of miR‐375/SLC7A11 axis on the progression of OSCC.

Though sufficient in vitro experiments were performed to study the functions of miR‐375/SLC7A11 on OSCC cells, the in vivo experiments should be supplemented to further confirm our results. And more targets of miR‐375 would be explored to figure out the related mechanism of OSCC progression.

In summary, our study indicated a negative targeting relationship between miR‐375 and SLC7A11. The expression of miR‐375 was downregulated in OSCC cells and such a conclusion is consistent with previous ones. Elevating the expression of miR‐375 inhibited the proliferation and invasion of OSCC cells through inhibiting the expression of SLC7A11. Replenishing of miR‐375 or silencing of SLC7A11 could be therapeutically valuable.

## Conflict of Interest

The authors indicated no potential conflicts of interest.

## Supporting information


**Figure S1.** The effects of miR‐375 mimics, SLC7A11 siRNA and pCDNA3.1‐SLC7A11 on cell viability were dependent on its transfected concentration.
