# Supplementary figures and images for: MiR‐375/SLC7A11 axis regulates oral squamous cell carcinoma proliferation and invasion
Source: Cancer Med. 2017 Jun 19;6(7):1686–97. doi: 10.1002/cam4.1110 (PMC5504333; doi:10.1002/cam4.1110)

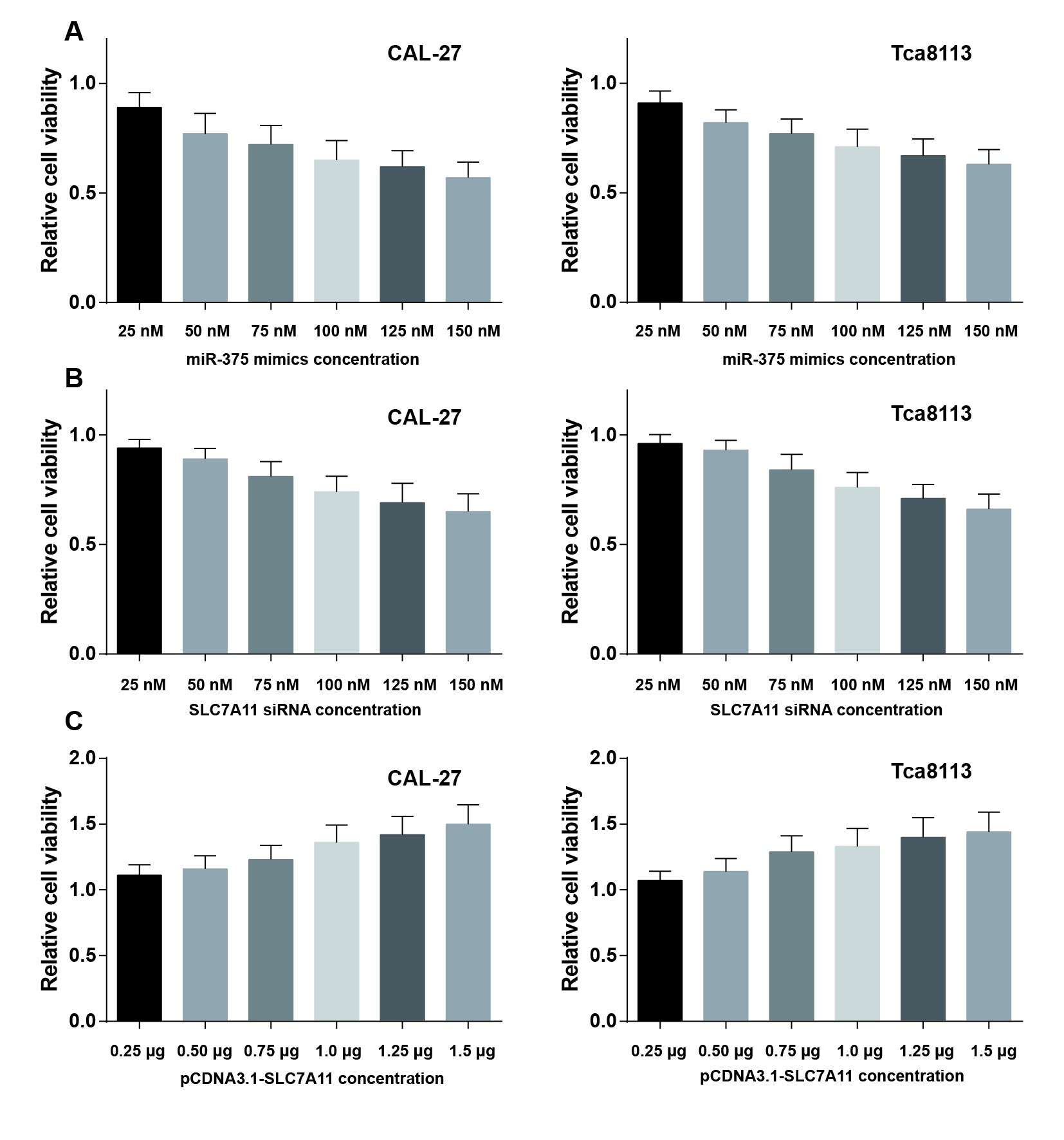

Supplement: Supplementary file 1 — Figure S1. The effects of miR‐375 mimics, SLC7A11 siRNA and pCDNA3.1‐SLC7A11 on cell viability were dependent on its transfected concentration. [file CAM4-6-1686-s001.tif]
